# An Outbreak of Parvovirus B19 in Israel

**DOI:** 10.3390/v15112261

**Published:** 2023-11-16

**Authors:** Tal Patalon, Yaki Saciuk, Daniel Trotzky, Gal Pachys, Amir Ben-Tov, Yaakov Segal, Sivan Gazit

**Affiliations:** 1 Kahn Sagol Maccabi (KSM) Research & Innovation Center, Maccabi Healthcare Services, Tel Aviv 68125, Israel; 2 Maccabitech Institute for Research and Innovation, Maccabi Healthcare Services, Tel Aviv 68125, Israel; 3 Shamir Medical Center (Assaf Harofeh Medical Center), Zerifin 70300, Israel; 4 Faculty of Medicine, Tel Aviv University, Tel Aviv 69978, Israel; 5 Maccabi Healthcare Services, Tel Aviv 68125, Israel

**Keywords:** human parvovirus B19, B19V, post-COVID-19 infectious diseases, social determinants of health

## Abstract

Human parvovirus B19 (B19V) has a wide clinical spectrum, ranging from an asymptomatic infection to a life threatening one. During pregnancy, it can lead to fetal loss and hydrops fetalis. This retrospective study examined the incidence rates of B19V in Israel, analyzing anonymized electronic medical records of 2.7 million individuals between January 2015 and September 2023. A generalized linear model with a Poisson distribution was fit to the data, adjusting for potential confounders. A marked increase in B19V was observed in 2023, with an adjusted incidence rate ratio (IRR) of 6.6 (95% CI 6.33–6.89) when comparing 2023 to previous years. When specifically comparing 2023 to COVID-19 years (2020–2022), adjusted IRR climbs to 9.21 (8.66–9.80). Moreover, in 2023, previously existing seasonality has largely disappeared. High SES characterized most infected individuals with a marked discrepancy in social sectors; the Arab population was significantly less likely to be found B19V positive, even when adjusting for SES. Most infections occurred in school-aged children (6–11 years old). Pregnant women experienced the most significant rise in B19V, with an adjusted IRR of 11.47 (9.44–13.97) in 2023 compared to previous years; most cases were diagnosed in the first trimester. This study demonstrates that Israel is currently experiencing the largest and longest reported outbreak of B19V to date. Policymakers should consider setting screening policies in place, at least for populations at risk, while specifically studying and potentially targeting low socioeconomic populations and specific social sectors to avoid health inequalities.

## 1. Introduction

Human parvovirus B19 (B19V) is a single-stranded DNA virus of the *Parvoviridae* family, known to be pathogenic for humans for over three decades [1,2,3,4]. It has a wide clinical spectra, ranging from asymptomatic infection, through to a mild disease and up to a life threatening one, largely depended on age, immunocompetence and pregnancy status. In children, most commonly school aged, B19V classically causes erythema infectiosum (fifth disease), a bi-phasic fever and rash illness. Erythema infectiosum is characterized by an initial non-specific flu-like prodromal phase of headache, sore throat, vomiting, diarrhea, fever and coryza [5,6]—attributed to B19V viremia—followed by the classical “slapped cheek” rash, an erythematous malar-like exanthem with circumferential pallor, two to five days later [7], corresponding to immune activation. The rash can later spread to the trunk and limbs, often as an erythematous reticular-type rash [8].

Other types of rashes have been associated with B19V, especially in adults, where the cutaneous manifestations are polymorphous [9], including gloves-and-socks involvement [10]. Nonetheless, joint involvement is the most common manifestation in individuals over 18 years, presented as polyarthralgia or polyarthritis in up to 60% of patients [11]. In fact, it has been previously suggested that as many as up to 15% of new arthritis cases can be attributed to B19V sequelae [6]. Arthropathy-related symptoms are usually symmetric, involve smaller extremity joints of the hands, wrists, knees, and feet [11,12,13], and resolve within a few weeks; though chronic manifestations have been previously described as well as case reports on its connection to other rheumatological diseases [13]. 

Transient Aplastic Crisis (TAC), a temporary suspension of red-blood cell production, is potentially a more serious manifestation of B19V, with severe anemia and related complications such as exacerbation of congestive heart failure, cerebrovascular events and acute splenic sequestration [14]. Interestingly, suppression of erythropoiesis seems to be present in the majority of B19V infections, evident by the reduction in reticulocytes, though anemia is not generally present, potentially due to rapid recuperation relative to erythrocytes life cycle [6]. However, in patients with hematological abnormalities, such as sickle cell disease, hemolytic anemia and hereditary spherocytosis, severe cases of TAC have been reported [7,14,15,16,17]. Other perilous complications include neurologic manifestations, reported across age groups, including both central nervous system complications, such as encephalitis, as well as peripheral ones as Guillain–Barré syndrome [18,19]. A large array of medical conditions have been linked to B19V, such as myocarditis, vasculitis, liver diseases, immune thrombocytopenia and others, but these have yet to be sufficiently established [6]. 

During pregnancy, B19V infection can lead to fetal loss, anemia and non-immune hydrops fetalis [4,6,20]. Rates of fetal loss vary between studies from around 10 percent [21,22,23], to 20–30 percent in earlier publications. While the risk exists throughout the pregnancy, it is significantly higher if infection occurred in the first trimester [24]. Fetal hydrops, or accumulation of excessive fluid in fetal compartments, is of greatest risk during the first and second trimesters, and contributes to the risk of fetal loss [3,4]. The transplacental transmission leads to a B19V infection of the fetal liver, the site of erythropoiesis, therefore leading to sever anemia, at times co-occurring with thrombocytopenia. Low hemoglobin contributes to high-output heart failure, though direct damage to the myocardium has also been described [20].

Lifetime prevalence of B19V is relatively high throughout the world; where genotypes 1 is prevalent worldwide, genotype 2 is rare and currently considered ancestral to genotype 1, while genotype 3 can be found primarily in Africa and South America; the intergenotype genetic divergence is up to 13% [25,26]. Prevalence increases with age and varies across geographic locations, with school age seroprevalence of 20–60% and up to 80% in adults in Western countries [27]. It is estimated that approximately 50–75% of women of reproductive age have been infected with B19V [28], while half of pregnant women are susceptible to infection [21,22]. In temperate climates a seasonal pattern is observed, with higher prevalence during late winter and early spring. Incidence can be sporadic, though peaks of large local outbreaks have been observed every few years [6,28]. In Israel, a single large-scale study was published on local epidemiology, dating ten years ago [27]. Mor et al. [27] found an overall national seroprevalence of 60%, with the last surge occurring in 2012. Since then, no significant increase in prevalence has been documented, though notably, there is no routine monitoring program in place. To this end, and given our collective clinical experience demonstrating a recent suspected rise in B19V encountered in our daily practice, we conducted a retrospective study, exploring the incidence rates of B19V among different population segments in recent years.

## 2. Materials and Methods

### 2.1. Data

#### 2.1.1. Data Sources

Maccabi Healthcare Services (MHS) is the second-largest non-for-profit health fund in Israel, covering 26.7% of the population (~2.7 million), in a national distribution, with less than 1% disengagement rate of its members. Membership in any of the four health funds is mandatory for all residents, and funds are prohibited from denying memberships. MHS has maintained a centralized database of electronic medical records (EMRs) for over three decades, allowing for a comprehensive longitudinal medical follow-up. This centralized dataset is comprised of extensive demographic data, clinical measurements, outpatient and hospital diagnoses and procedures, medications dispensed, imaging performed and comprehensive laboratory data from a single central laboratory.

#### 2.1.2. Data Extraction and Study Population

Anonymized EMRs were retrieved from MHS, where data of all members between 1 January 2015 through 30 September 2023 were extracted. Individual-level data included demographics, namely sex, age, area of residence, social sector and socioeconomic status (SES). The latter is assigned by the Israel’s Central Bureau of Statistics [29], and is measured on a scale of 1 (lowest) to 10, where index is calculated based on several parameters, including household income, educational qualifications, household crowding and car ownership. Data also consisted of immunocompetence, based on inclusion in MHS’ automated registry of immunosuppression [30], as well as of pregnancy status. B19V-related data included any coded diagnosis—ICD9 079.83 (Parvovirus B19) and 057.0 (Erythema infectiosum (fifth disease)), or their corresponding internal MHS codes—inpatient or outpatient, as well as anti-B19V serology results, performed at MHS central laboratory. 

### 2.2. Study Design and Statistical Analysis

A retrospective cohort study, examining incidence rates of B19V between 2015 and 2023. A B19V incidence case was defined as either a positive IgM test performed at MHS’ central laboratory or a B19V diagnosis code during the study period. We included both laboratory tests and diagnoses codes for two main reasons: first, in younger populations, diagnosis is largely based on clinical symptoms, often not necessitating serological confirmation [31]; second, in more severe cases, diagnosis is performed at an inpatient setting, and in such a scenario, laboratory results might not be fully reported back to the outpatient MHS setting, while a diagnoses code will be.

We used a non-adjusted incidence rate per 10,000 person days at risk to demonstrate rates over time, by month. Next, a generalized linear model (GLM) with a Poisson distribution was fit to the data [32], adjusting for possible confounders including SES, social sector, district of residence, age (using splines), sex and calendar month (for seasonality). Then, we calculated the incidence rate ratio (IRR), specifically comparing the incidence rate of B19V in 2023 to that of previous years, where the latter were the reference group. Additionally, we compared 2023 to COVID-19 years (2020–2022) and pre-COVID-19 years (2015–2019), in order to assess IRR of two different reference points in terms of social and health-care-related behavior.

We further analyzed the documented B19V data by age groups, focusing on children and adolescents, stratifying the population into three non-overlapping groups which match educational stages, which has been previously shown to affect exposure as well: newborn to age 6 (not inclusive), 6 to 12 years (primary school) and adolescents aged 12 to 18 years (junior high and high school). Additionally, we separately examined pregnant women, stratifying analysis by the trimester in which the woman was first infected.

Additionally, we conducted two supplementary analyses. The first one included a sensitivity analysis, where a repeated Poisson analysis was performed, recalculating IRRs, separating the case definition of B19V by serology only and by diagnosis only. The second supplementary analysis included an examination of cytomegalovirus (CMV) as a negative outcome control [33], comparing incidence in 2023 to the previous study years (2015–2022), implementing a logistic regression analysis, adjusting for the same variables as the primary analysis. CMV-positive individuals included all MHS members with at least one positive CMV-IgM serology result during the study period. To avoid misclassification, patients with borderline CMV-IgM testing or a concurrent positive Epstein–Barr (EBV)-IgM results were excluded, following a previously published diagnostic algorithm based on our database [34]. The rationale was to examine whether an uncontrolled confounder is involved in the suspected and hypothesized recent rise in B19V, such as healthcare utilization or practices.

All analyses were conducted using R Studio version 3.6. The analysis conforms to Strengthening the Reporting of Observational Studies in Epidemiology (STROBE) Statement.

### 2.3. Ethics and Data Declarations

The study was approved by the Maccabi Healthcare Services (MHS) Institutional Review Board (IRB). Informed consent was waived by the IRB, and all identifying details of the participants were removed prior to analysis.

## 3. Results

### 3.1. Primary Analysis: Incidence and Trend in the General Population

**Figure 1:**
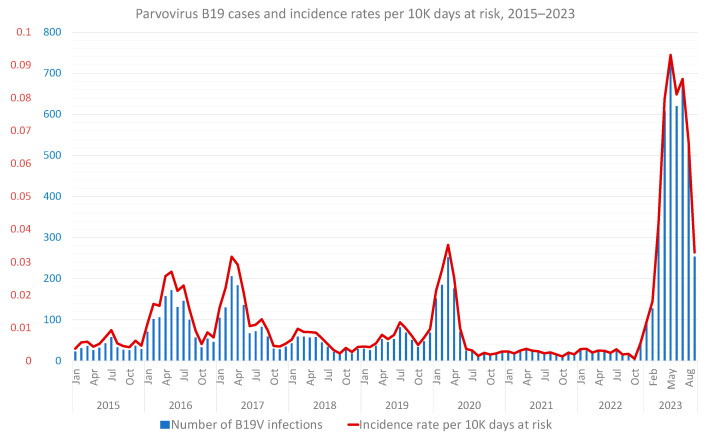
Parvovirus B19 incidence rates per 10,000 person days at risk between 2015 and 2023, by month.

A total of 9178 B19V infections have been documented in MHS since January 2015, with a marked increase starting in 2023 (Figure 1), when more than 40% of the total number of infections during the nine investigated years were documented. Up to 2023, three seasonal peaks were recorded, between winter and spring to early summer, during 2017, 2018 and 2020. However, in 2023, seasonality has largely disappeared, with a marked and continuous surge in incidence occurred carried into late summer and early autumn. September 2023, however, which accounted for the last month of follow-up, exhibited an apparent downslope in B19V rates, though these are still markedly higher than previous autumns.

**Figure 2:**
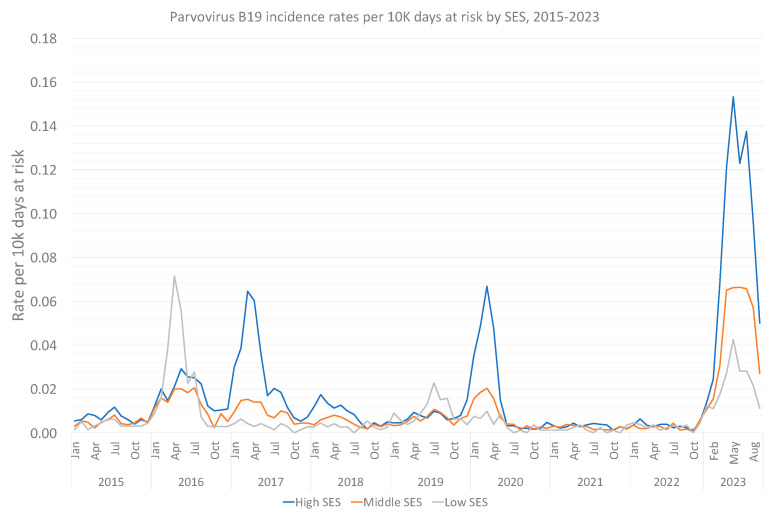
Monthly Parvovirus B19 incidence rates per 10,000 person days at risk between 2015 and 2023, by month, by socioeconomic status.

When particularly examining the characteristics of the 2023-infected population (Table 1), the younger population prevails with a median age of 7.33 (0, 79.6), alongside a female dominance (58.6%) among B19V-positive individuals. No clinically important difference in immunosuppression rates was found between infected and uninfected individuals. High SES characterized most infected individuals in 2023, and throughout most of the 9-year study period (Figure 2). A marked discrepancy in the social sector was found, with Arabs and Orthodox Jewish individuals having less reported B19V infections, compared to their relative representation in the total population.

Further validating the existence of an outbreak, the Poisson regression analysis demonstrated an IRR of 7.07 (95% CI, 6.78–7.37) when comparing 2023 B19V-infected individuals to those between 2015 and 2022 (Table 2). IRR remained relatively unchanged (6.6, 95% CI, 6.33–6.89) even when adjusting for sex, age, residential district, social sector, SES and calendar month. When specifically comparing 2023 to COVID-19 years (2020–2022), adjusted IRR climbs to 9.21 (95% CI, 8.66–9.80), versus 5.47 (95% CI, 5.23–5.73) when comparing 2023 to pre-COVID years (2015–2019). Lastly, given the potential overlap between SES and affiliation to the ultra-orthodox Jewish or Arab populations, we conducted a dedicated Poisson regression analysis (Table 3). It appears that the Arab population is significantly less likely to be found B19V positive, even when adjusting to SES.

**Figure 3:**
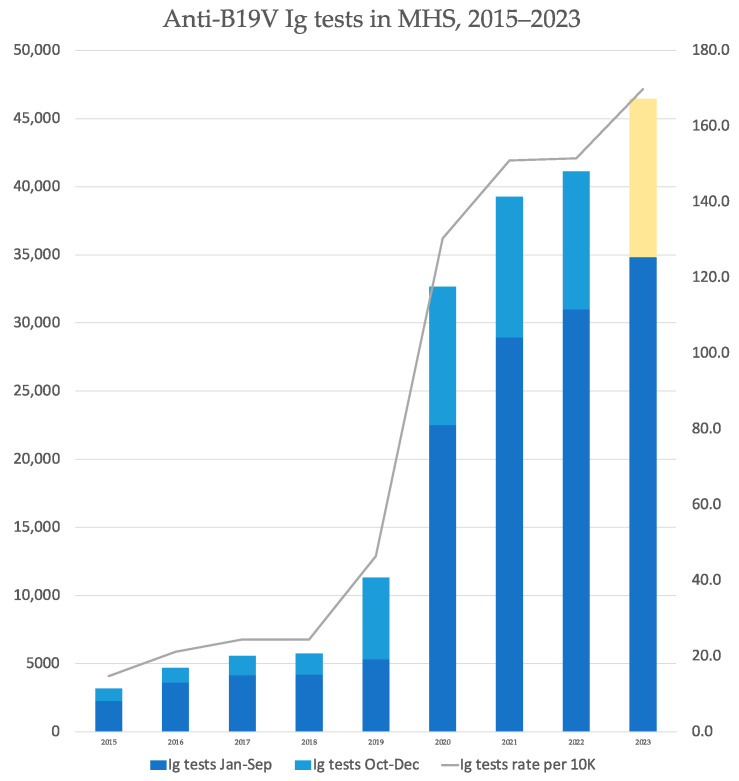
Number of Parvovirus B19 serology tests taken each year, and their respective rates per 10,000 person days at risk. To allow for seasonality considerations, months October to November are separated visually, as the corresponding data in 2023 is not included. A linear extrapolation of this quarter is present in 2023 is presented in yellow.

Examining the overall number of serology tests performed in the community setting, we found an increase in 2019, with testing rates per ten thousand MHS members almost doubling from 24.30 to 46.37, a phenomenon that repeated itself in the following year (Figure 3). A constant, though milder, increase in testing rates continued throughout 2021–2023. Nevertheless, when comparing IgM positivity percentages, it is evident that the surge of cases in 2023 is accompanied by a respective surge of positivity rates, without a significant corresponding increase in the overall testing rates (Table 4).

### 3.2. Sub-Analysis: Children and Pregnant Women

**Figure 4:**
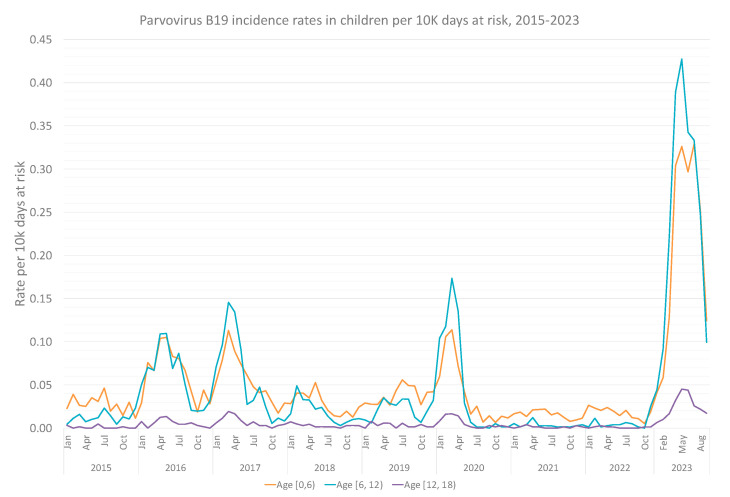
Monthly Parvovirus B19 incidence rates per 10,000 person days at risk of MHS member under 18 years between 2015 and 2023, by age group.

Overall, 7633 infections of children and adolescents were recorded between 2015 and 2023 (83% of the overall infections), with most infections occurring in school-aged children (6–11 years old), followed by the 0–5 years population. Infections of adolescents 12–17 years old were rare (Figure 4). Table 2 presents the Poisson regression analysis, with adjusted and unadjusted rate ratios for each age-group sub-cohort. Even more prominently than in the general population, the recent year, compared to the previous eight ones, marks a surge in B19V infections in school-aged children, with an adjusted IRR of 7.72 (95% CI, 7.22–8.24).

**Figure 5:**
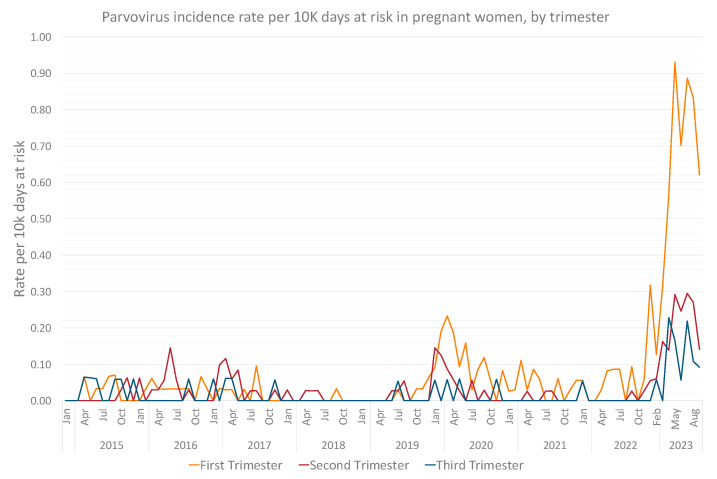
Monthly Parvovirus B19 incidence rates per 10,000 person days at risk of pregnant women between 2015 and 2023, by trimester of infection.

Pregnant women experienced the most significant rise in B19V incidence, with an adjusted IRR of 11.47 (95% CI, 9.44–13.97) in 2023 compared to previous years (Table 2). Most cases were diagnosed in the early gestation, with peak infection rates of 0.93, 0.29 and 0.23 per ten thousand person days at risk, in the first, second and third trimester, respectively (Figure 5).

### 3.3. Supplementary Analyses

In the first supplementary analysis, rerunning the Poisson regression analysis while separating the confirmatory vertical of B19V cases (diagnosis versus serology) yielded similar results, as can be observed in Table 5. In the second supplementary analysis, we found that the adjusted Odds Ratio (aOR) for CMV infection in 2023 (January to September) compared to 2015–2022 was 0.965 (95% CI, 0.933–0.997), demonstrating no actual change in CMV infection trends in 2023.

## 4. Discussion

To the best of our knowledge, this is the largest and longest-spanning study of B19V in Israel to date, analyzing 9 years of data, including over 2.7 million individuals. Our retrospective design calculated the monthly rates of B19V infection, while additionally specifically comparing the current year to all previous ones.

Our analysis demonstrated a surge of B19V cases in 2023 compared to previous years, with over 40% of the total number of infections in the past nine years occurring during the last nine months. Correspondingly, the rate ratio for a B19V infection in 2023 compared to 2015–2022 was 6.6 (95% CI, 6.33–6.89) when adjusting for sex, age, SES, district of residence, social sector and calendar month. Although B19V incidence has been known to peak every few years [6,28], the current surge is the highest known to date. Indeed, the demonstrated peak in 2023 not only represents the highest incidence rates in the nine examined years of our study, including in comparison to previous mild surges within that time, but rather the highest-ever published incidence rate in Israel. One previous study [27] reported two surges in 2008 and 2011–2012, though absolute number of positive cases were much smaller, while positivity rates were less informative as they represent a limited data source. Interestingly, when comparing 2023 to peak COVID-19 years, the surge is even more pronounced, with a 9-fold increase in incidence rates; a much steeper rise than compared to pre-COVID years. It has been suggested that COVID-19 has led to changed patterns of other viral illnesses, in children especially, potentially due to social and healthcare-related behaviors leading to an immune gap [35]. This has been demonstrated specifically for B19V as well, with studies indicating lower incidence during the pandemic [36,37,38,39].

Apart from its larger scope, the current outbreak also influenced altered previous seasonality patterns; in the preceding two surges, peaks were documented between winter to early summer, following the known pattern of temperate climates [6]; however, in 2023, the highest incidence rates were continuous and carried into the autumn months. September, the last month of follow-up, represents an apparent slowing down of the surge but incidence rates are still higher than previous fall seasons. Further studies and continuous follow-up are needed to conclusively determine seasonality.

As expected, children make up the largest age group of documented infections in absolute numbers, representing over 80% of overall infections during the nine studied years, as well as in the current surge, characterized by a median age of 7.3 of infected individuals. School aged children (ages 6–12) made up the most detected population of individuals under 18, with an adjusted IRR of 7.72 (95% CI, 7.22–8.2 [4]) of B19V in 2023 compared to this age group in previous years. The relative high representation of this age group could be potentially explained by classic symptomatology of erythema infectiosum [5,6] and corresponding awareness of clinicians, testing and diagnosing B19V.

A similar rationale, of increased awareness due to potential fetal complications, could partially account for the relative increase in infection rates in pregnant women, with an IRR of 11.47 (95% CI, 9.44–13.97) in 2023 compared to previous years, mostly in the first trimester; this rationale has been suggested in other studies as well [40]. Though up to 80% of adults will have been exposed to B19V during their lifetime, the existing literature suggests that half of pregnant women are susceptible to infection [21,22]. Importantly, it has been demonstrated that during surges, secondary attack rates are significant [28], as has been demonstrated by our data as well. Rates have also been shown to be highly dependent on occupation deriving substantial exposure opportunities, such as daycare center workers or schoolteachers, among whom infection rates of susceptible women can climb to 20 to 30 percent [28], while hydrops fetalis and fetal death have also been demonstrated to rise during outbreaks [25,41]. Increased rates in this population could also be related to secondary transmission in households, a hypothesis that should be researched in future studies.

Our analysis is subject to limitations. First, as there is no mandatory or routine screening policies for B19V in Israel, and given the potential asymptomatic presentation—in pregnancy as well [4]—incidence could be much higher. Second, as children and women of in early gestational stages are more likely to be sent for testing (and given the lack of a generalized or controlled serology practices), the relative incidence between groups (i.e., pregnant vs. non-pregnant, children vs. adults) cannot be sufficiently assessed. Additionally, our data pointing to high SES as characterizing B19V infections suggests discrepancies in practice and tests offered and emphasized the importance of social determinants of health. This is also the case for the Arab population, which was significantly less likely to be found B19V positive, even when adjusting to SES. Finally, a reasonable concern in analyzing infectious diseases in absence of regulated screening is that the increased incidence merely represents an increased testing practice. However, no new testing policies have been issued in 2023, and as demonstrated, an increase in serology testing started in 2019, not in 2023. Moreover, a corresponding surge of positivity rates, without a significant rise in overall testing rates, characterized 2023. Moreover, especially in children, the presentation of erythema infectiosum is clinically quite specific and is less dependent on testing [31]. Lastly, our supplementary analysis of CMV as a negative outcome control demonstrated no such rise in 2023, further strengthening the B19V specific observed surge.

## 5. Conclusions

In conclusion, our study demonstrates that Israel is currently experiencing the largest and longest reported outbreak of B19V to date, with a significant increase seen in both school-aged children and pregnant women. Policymakers should consider setting screening policies in place, at least for populations at risk, while specifically studying and potentially targeting low socioeconomic populations and specific social sectors to avoid health inequalities.

## Figures and Tables

**Table 1:** Characteristics of MHS participants in 2023, with and without a history of Parvovirus B19 infection. Data represent number (%) of participants, unless stated otherwise.

	No	Yes	Overall
	**(*N* = 2,747,624)**	**(*N* = 3977)**	**(*N* = 2,751,601)**
**Sex**			
Female	1,401,306 (51.0%)	2331 (58.6%)	1,403,637 (51.0%)
Male	1,346,318 (49.0%)	1646 (41.4%)	1,347,964 (49.0%)
**Age**			
Mean (SD ^1^)	34.9 (23.0)	12.2 (13.1)	34.8 (23.0)
Median [Min, Max]	32.4 [0.0, 102]	7.33 [0.0, 79.6]	32.4 [0.0, 102]
**SES ^2^**			
High (8–10)	927,084 (33.7%)	2090 (52.6%)	929,174 (33.8%)
Middle (5–7)	1,491,338 (54.3%)	1703 (42.8%)	1,493,041 (54.3%)
Low (1–4)	322,550 (11.7%)	180 (4.5%)	322,730 (11.7%)
Other	6652 (0.2%)	4 (0.1%)	6656 (0.2%)
**Social Sector**			
Jewish	2,291,679 (83.4%)	3622 (91.1%)	2,295,301 (83.4%)
Arab	175,545 (6.4%)	27 (0.7%)	175,572 (6.4%)
Orthodox Jewish	280,400 (10.2%)	328 (8.2%)	280,728 (10.2%)
**Immunocompromised**			
No	2,738,155 (99.7%)	3971 (99.8%)	2,742,126 (99.7%)
Yes	9469 (0.3%)	6 (0.2%)	9475 (0.3%)

^1^ SD—standard deviation; ^2^ SES—socioeconomic status.

**Table 2:** Incidence rate ratio of Parvovirus B19 in 2023 compared to 2015–2022 based on a Poisson regression analysis for each studied population group. Right column demonstrated the adjusted incidence rate ratio per 100,000 person days, accounting for sex (when applicable), age (using splines), socioeconomic status, residential district, social sector and calendar month. For each column, the 95% confidence intervals are presented in parenthesis.

Population	IRR ^1^ of B19V ^2^ Incidence in 2023 Compared to 2015–2022	Adjusted IRR ^1^ of B19V ^2^ Incidence in 2023 Compared to 2015–2022
Total population	7.07 (6.78, 7.37)	6.6 (6.33, 6.89)
All Children	7.16 (6.84, 7.49)	6.39 (6.1, 6.69)
Children aged [0, 6)	5.92 (5.53, 6.34)	5.24 (4.9, 5.62)
Children aged [6, 12)	8.73 (8.18, 9.31)	7.72 (7.22, 8.24)
Children aged [12, 18)	7.21 (5.9, 8.81)	6.2 (5.07, 7.58)
Pregnant women	12.15 (10.06, 14.69)	11.47 (9.44, 13.97)

^1^ IRR—incidence rate ratio; ^2^ B19V—Human parvovirus B19.

**Table 3:** Incidence rate ratio of Parvovirus B19 infection per 100,000 person days in 2015–2023 by social sector, adjusted for sex, age (using splines), residential area and calendar month. For each column, the 95% confidence intervals are presented in parenthesis.

Variable	Category	Adjusted IRR ^1^
Social sector		
	Jewish	Ref
	Arab	0.29 (0.24, 0.35)
	Ultra-orthodox	0.98 (0.90, 1.06)

^1^ IRR—incidence rate ratio.

**Table 4:** Number of overall serology tests (IgG and IgM) for Parvovirus B19 performed during the study period with proportion of positive IgM test, by year.

Year	Total Serology Tests	Positive IgM Tests	Proportion (%) of Positive IgM Tests out of Total Performed Tests
2015	3180	112	3.5%
2016	4682	268	5.7%
2017	5560	248	4.5%
2018	5738	68	1.2%
2019	11,314	80	0.7%
2020	32,650	207	0.6%
2021	39,275	52	0.1%
2022	41,129	64	0.2%
2023	34,838	1053	3.0%

**Table 5:** Supplementary analysis. Incidence rate ratio of Parvovirus B19 in 2023 compared to 2015–2022 based on a Poisson regression analysis for each studied population group, separated by source of B19V confirmation, diagnosis or serology. Right column demonstrated the adjusted incidence rate ratio per 100,000 person days, accounting for sex (when applicable), age (using splines), socioeconomic status, residential district, social sector and calendar month. For each column, the 95% confidence intervals are presented in parenthesis.

	**Serology Confirmed**		**Diagnosis Confirmed**	
**Population**	**IRR** **^1^ of B19V** **^2^ Incidence in 2023** **Compared to 2015–2022**	**Adjusted IRR** **^1^ of B19V** **^2^ Incidence in 2023** **Compared to 2015–2022**	**IRR** **^1^ of B19V** **^2^ Incidence in 2023** **Compared to 2015–2022**	**Adjusted IRR** **^1^ of B19V** **^2^ Incidence in 2023** **Compared to 2015–2022**
Total population	9.43 (8.60, 10.34)	8.51 (7.73, 9.36)	6.89 (6.60, 7.20)	6.48 (6.19, 6.78)
All Children	7.18 (6.02, 8.55)	6.21 (5.19, 7.44)	7.23 (6.91, 7.57)	6.45 (6.16, 6.77)
Children aged [0,6)	6.65 (4.66, 9.41)	5.77 (4.04, 8.18)	5.96 (5.56, 6.38)	5.28 (4.92, 5.66)
Children aged [6,12)	7.44 (5.97, 9.26)	6.45 (5.16, 8.06)	8.88 (8.31, 9.49)	7.86 (7.35, 8.40)
Children aged [12,18)	7.00 (4.10, 11.80)	5.93 (3.46, 10.01)	7.27 (5.90, 8.94)	6.25 (5.08, 7.69)
Pregnant women	13.03 (10.65, 15.97)	12.73 (10.33, 15.74)	10.85 (8.34, 14.12)	10.13 (7.74, 13.31)

^1^ IRR—incidence rate ratio; ^2^ B19V—Human parvovirus B19.

## Data Availability

According to the Israel Ministry of Health regulations, individual-level data cannot be shared openly. Specific requests for remote access to de-identified community-level data to or to the code used for data analysis should be referred to KSM, Maccabi Healthcare Services Research and Innovation Center.
